# Effects of l-carnitine Administration on Sperm and Sex Hormone Levels in a Male Wistar Rat Reproductive System Injury Model in a High-Altitude Hypobaric Hypoxic Environment

**DOI:** 10.1007/s43032-022-00948-5

**Published:** 2023-01-12

**Authors:** Dehui Chang, Feiyan Kong, Wei Jiang, Fudong Li, Chunlei Zhang, Haoshuai Ding, Yindong Kang, Weiping Li, Chuang Huang, Xin Zhou, Xiaoli Zhang, Hongmei Jiao, Yafen Kang, Xuejun Shang, Bin Zhang

**Affiliations:** 1 Department of Urology, The 940th Hospital of PLA Joint Logistics Support Force, Lanzhou, Gansu China; 2 Second Department of Surgery, Beijing Fengtai Hospital of Integrated Traditional Chinese and Western Medicine, Beijing, China; 3 Air Force Hangzhou Secret Service Rehabilitation Center, Convalescent Section First of Convalescent Zone Second, Hangzhou, Zhejiang China; 4 The First Affiliated Clinical Medical College, Gansu University of Traditional Chinese Medicine, Lanzhou, Gansu China; 5 Department of Urology, Jinling Hospital, School of Medicine, Nanjing University, Nanjing, Jiangsu China

**Keywords:** l-carnitine, Male, Plateau, Environment, Reproductive damage, Injury model

## Abstract

The plateau environment impacts male reproductive function, causing decreased sperm quality and testosterone levels. l-carnitine can improve the semen microenvironment. However, the role of l-carnitine in a high-altitude environment remains unclear. In our study, we investigated the effects of l-carnitine administration in a male Wistar rat reproductive system injury model in the context of a simulated high-altitude environment. Rats were randomly divided into a normal control group (group A1, A2-low dose and A3-high dose) and high-altitude model groups (group B, C-low dose and D-high dose) with 20 rats in each group. With the exception of the normal control group exposed to normoxic conditions, the other groups were maintained in a hypobaric oxygen chamber that simulated an altitude of 6000 m for 28 days. In the experimental period, the low-dose groups (A2 and C) were administered 50 mg/kg l-carnitine via intraperitoneal injection once a day, and the high-dose groups (A3 and D) were given 100 mg/kg. After the feeding period, blood samples were collected to assess blood gas, serum hormone levels and oxidative stress. Sperm from the epididymis were collected to analyse various sperm parameters. After obtaining the testicular tissue, the morphological and pathological changes were observed under a light microscope and transmission electron microscopy (TEM). The impact of the simulated high-altitude environment on the rat testis tissue is obvious. Specifically, a decreased testicular organ index and altered indices of arterial blood gas and serum sex hormone levels caused testicular tissue morphological damage, reduced sperm quality, increased sperm deformity rate and altered malondialdehyde (MDA), superoxide dismutase (SOD) and glutathione peroxidase (GSH-Px) concentrations. The results demonstrate that l-carnitine can be administered as a preventive intervention to reduce the reproductive damage caused by high-altitude hypobaric and hypoxic environments and improve semen quality in a rat model.

## Introduction

The thin oxygen, low oxygen partial pressure, strong ultraviolet rays, large temperature differences between day and night and long sunshine hours in plateau areas represent unique climate characteristics that can cause many adverse effects on the human body [1–3]. In recent years, the impact of the plateau environment on male reproductive health has received attention, but there is limited research on the male reproductive system in plateau hypoxic environments [4, 5]. Although some studies have demonstrated that a high-altitude hypoxic environment can cause damage to male testicular reproductive function and negatively affect male semen quality, the specific mechanism is not completely clear. Oxidative stress is one of the main reasons for the damage of high-altitude environmental factors to the body, which leads to excessive production of reactive oxygen species (ROS). This is also one of the main molecular causes that contributes to varicocele-related male infertility [6, 7]. In addition, research on how to avoid or reduce high-altitude environment damage to the male reproductive system is available [6, 7].

At present, there are a few experimental studies that have used medical laboratory animals placed in a hypoxic environment to simulate acute altitude sickness [6, 8]. Therefore, the establishment of a high-altitude hypobaric hypoxic reproductive system injury model is one of the key links in the research of plateau-related reproductive system diseases [4, 9]. l-Carnitine exhibits good utility in maintaining sperm function, enhancing sperm motility and improving epididymal function, and has been maturely used in clinical treatment due to its safety and efficacy [10, 11]. Interestingly, a high concentration of carnitine is found in the male reproductive tract, especially in the epididymis, suggesting its crucial role in energy metabolism and spermatozoa maturation [11]. However, there are very few reports in the literature on the protection provided by the application of l-carnitine to reduce the damage to the male reproductive system in the plateau environment [12].

In this study, a high-altitude hypobaric and hypoxic Wistar male rat model was constructed to explore the impact of a high-altitude environment on the male reproductive system and discover the mechanism of l-carnitine on the male reproductive system in a plateau environment.

## Materials and Methods

### Experimental Animals and Groups

In our study, we raised 120 specific pathogen-free (SPF) grade Wistar male rats (180 ± 20 g weight and 24 weeks old, which is equivalent to 18–20 years old in humans) provided by the Experimental Animal Center of the 940th Hospital of Joint Logistics Support force of Chinese People’s Liberation Army (licence: SCXY (Military) 2012–0020). The rats were randomly divided into 4 groups (each group of 20 rats): group A1 (normal control group under normoxia), group A2 (normal control group under normoxia, received low-dose l-carnitine at 50 mg/kg), group A3 (normal control group under normoxia, received high-dose l-carnitine at 100 mg/kg), group B (plateau model control group, received injecting normal saline), group C (low-dose l-carnitine intervention altitude model group, received 50 mg/kg l-carnitine) and group D (high-dose l-carnitine intervention altitude model group, received 100 mg/kg l-carnitine).

#### Feeding Conditions

The automatic feeding system added food to the rats. The environmental temperature was 22 ~ 24 °C. The simulated time was 12 h. Groups A1–3 were raised in the clean room of the out-of-compartment animal centre and treated under normoxic conditions. The normoxic and hypoxic feeding conditions were the same except for the simulated altitude. The other groups were placed in a low-pressure environment simulated at 6000-m altitude in a low-oxygen chamber. l-Carnitine and normal saline were administered by intraperitoneal injection once a day for 28 days in total (2 rat’s spermatogenic cycles). Rat feed was provided by Beijing Keyao Xieli Feed Co., Ltd., and the product code is 2012/2252. Rat diet ingredients (g/kg) are given in Table 1 (all ingredients total 1000 g).

**Table 1:** Rat diets formulated for growth, maintenance and long-term tests

Ingredient	
	g/kg diet
Casein, 30 mesh	200.000
l-Cystine	3.000
Cornstarch	397.486
Maltodextrin 10	132.000
Sucrose	100.000
Cellulose	50.000
Soybean oil	70.000
t-Butylhydroquinone	0.014
Mineral mix S10022M	35.000
Vitamin mix V10037	10.000
Choline bitartrate	2.500

### Animal Ethics and All Procedures

Ethics approval was provided by the Animal Ethics Committee of Joint Service Support Force 940 Hospital of PLA. The experiment met the requirements of animal ethics with IRB no. 2021KYLL032 and IACUC Approval no. 2021LQBZ015 provided by the Institutional Animal Care and Use Committee of Joint Service Support Force 940 Hospital of PLA.

The main experimental equipment and reagents are shown in Table 2.

**Table 2:** Experimental equipment and reagents

Experimental equipment	
Simulated plateau low-pressure and hypoxic animal experimental chamber	AVIC Guizhou Fenglei Aviation Machinery Co., Ltd
TG16-WS desktop high-speed centrifuge	Hunan Xiangyi Laboratory Instrument Development Co., Ltd
XT-2000i animal whole blood cell analysis instrument	Japan Sysmex Medical Electronics Co., Ltd
Optical microscope	Phoenix Optical Group Co., Ltd
Sperm counting plate	Shanghai Weitu Instrument Technology Development Co., Ltd
37 °C constant temperature box oscillator	Shanghai Yiheng Technology Co., Ltd
TG16-WS desktop high-speed separation	Hunan Xiangyi Laboratory Instrument Development Co., Ltd
Experimental reagents	
MDA Kit	Nanjing Jiancheng Technology Co., Ltd
SOD Kit	Nanjing Jiancheng Technology Co., Ltd
GSH-Px Kit	Nanjing Jiancheng Technology Co., Ltd

### The Establishment of the Rat Reproductive System Injury Model of Hypobaric Cabin Simulating High Altitude

The low-pressure and hypoxic animal experimental cabin could simulate a plateau environment and was used to set different altitudes for animal experiments. Groups B, C and D were placed in this low-pressure cabin, whereas groups A1–3 were not. The door was sealed. An automatic electronic vacuum pump was used to extract part of the air (the simulated altitude decompression speed of 2 m/s) in the cabin to simulate the plateau (altitude of 6000 m). Fresh air circulation was provided for 4–6 h (to ensure a stable pressure in the cabin during the ventilation process), and the temperature was maintained between 22–24 °C in the cabin. The rats were free to eat and drink using the automatic feeding system for 28 days (2 spermatogenic cycles in rats). After 28 days, the experiment was completed according to the design. The intake valve was adjusted, and the simulated environment was returned to the normal altitude (the simulated altitude compression speed was 2 m/s).

### Blood Gas Measurement

After the experiment, the rats in each group were removed from the cabin and anaesthetized by intraperitoneal injection of 10% chloral hydrate at a dose of 1 ml/kg. The abdominal aorta was isolated after exposing the abdominal cavity in the supine position. One millilitre of arterial blood was placed into a heparin anticoagulation tube, and blood gas analysis was performed within 5 min.

### Sex Hormone Levels

Other blood samples were collected from the abdominal aorta of the rats and placed into coagulation tubes. Tubes were placed at room temperature to allow the blood to coagulate naturally. Then, the blood samples were centrifuged, and the serum was collected. The levels of serum testosterone (T), luteinizing hormone (LH) and follicle-stimulating hormone (FSH) in rat serum were detected by using an enzyme-linked immunoassay (ELISA). The procedure was performed according to the requirements of the manual test kit.

### Testicular Tissue Extraction

The rat testicular tissues were stripped, and the general pathological changes in the testicular tissue were observed. The right testicular tissues were obtained and placed in electron microscope fixation solution, washed with phosphate buffer, and then fixed in 1% osmic acid·0.1 M phosphate buffer. After rinsing and dehydrating with an ethanol gradient, the tissues were embedded in the sections and placed under TEM for observation. After splitting the right testis longitudinally, the tissues were fixed in 10% formaldehyde for 24 h, and other tissues were removed for the assessment of malondialdehyde (MDA) and superoxide dismutase (SOD) levels. The tissues were fixed with 10% formaldehyde for 24 h; embedded in paraffin; cut into 4- to 6-μm sections; mounted on clean glass slides; conventionally deparaffinized, dehydrated and mounted after haematoxylin–eosin (HE) staining and then observed under a light microscope.

### Detection of Sperm Parameters

The epididymal tails of rats from each group were obtained. Tissues were washed with PBS (pH = 7.2) first, cut by ophthalmological scissors and shaken in 2 ml PBS evenly. The samples were allowed to stand for 20 min and then filtered. After letting the samples stand for 3 min again, the supernatants were collected to prepare sperm suspensions for the detection of sperm viability rate, sperm count and sperm deformity rate. In this study, assessment of the sperm viability rate, sperm count and sperm deformity rate was performed manually. Papanicolaou staining was used for the sperm deformity rate. Papanicolaou stain clearly stains the acrosome region of the sperm head, excess residual cytoplasm and the terminal and main segments. Sperm processing was based on the WHO(World Health Organization)laboratory manual for the examination and processing of human semen (the Fifth Edition, February 2011).

### Detection of MDA, SOD and GSH-Px Levels

This procedure must be performed on the icebox in accordance with the manual instructions. Portions of right testis tissues were removed, rinsed, weighed and shredded. The tissues were made into homogenates. The prepared 10% homogenate was centrifuged, and the supernatants were obtained and saved for subsequent detection experiments.

### Statistical Processing

We used SPSS 20.0 statistical software for the data statistics. Quantitative data conforming to a normal distribution and homogeneity of variance were expressed as ($\overline{x} \pm s$). Using factorial design variance analysis, we compared the differences in the levels of blood gas, sperm motility, sperm count, deformity rate, MDA, SOD and GSH-Px levels from groups at different altitudes. Using single-factor analysis of variance, we compared the means of multiple samples, and the *t* test was used to compare the means of two samples. *P* < 0.05 indicates that the difference was statistically significant.

## Results

### Testicular Organ Index of Rats

**Fig. 1:**
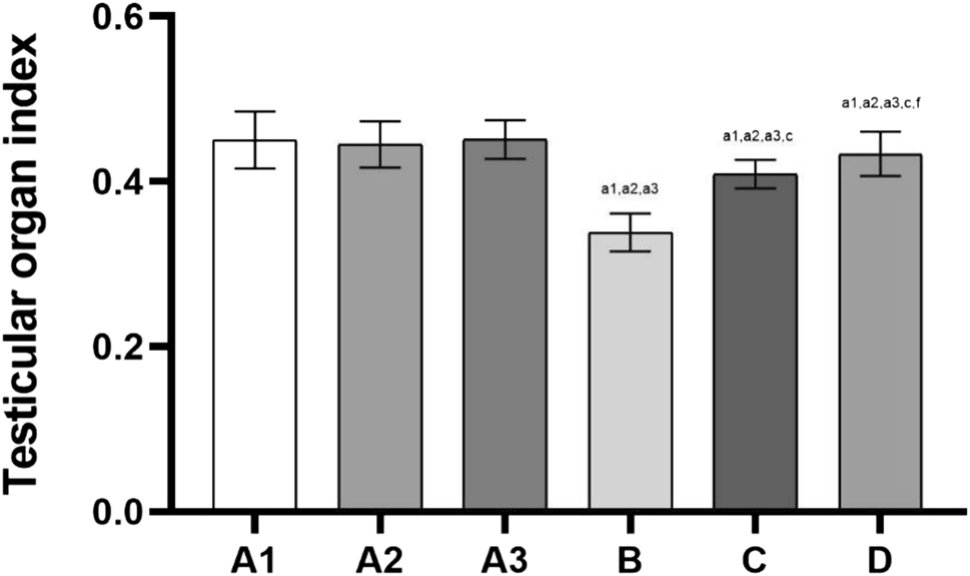
Testicular organ index of rats in each group. Note: Compared with group A1, a1: *P* < 0.01, b1: *P* < 0.05; Compared with group A2, a2: *P* < 0.01, b2: *P* < 0.05; compared with group A3, a3: *P* < 0.01, b3: *P* < 0.05; compared with group B, c: *P* < 0.01, d: *P* < 0.05; compared with group C, e: *P* < 0.01, f: *P* < 0.05

The test results are shown in Fig. 1.

The results of the testicular organ index were not significantly different among groups A1, 2 and 3 (*P* > 0.05).

Compared with groups A1, 2 and 3, the indices were all decreased in groups B, C and D (*P* < 0.01, and the difference was statistically significant).

Compared with group B, the indices were increased in groups C and D (*P* < 0.01, and the difference was statistically significant).

Compared with group C, the indices were increased in group D (*P* < 0.05, and the difference was statistically significant).

### Arterial Blood Gas

**Fig. 2:**
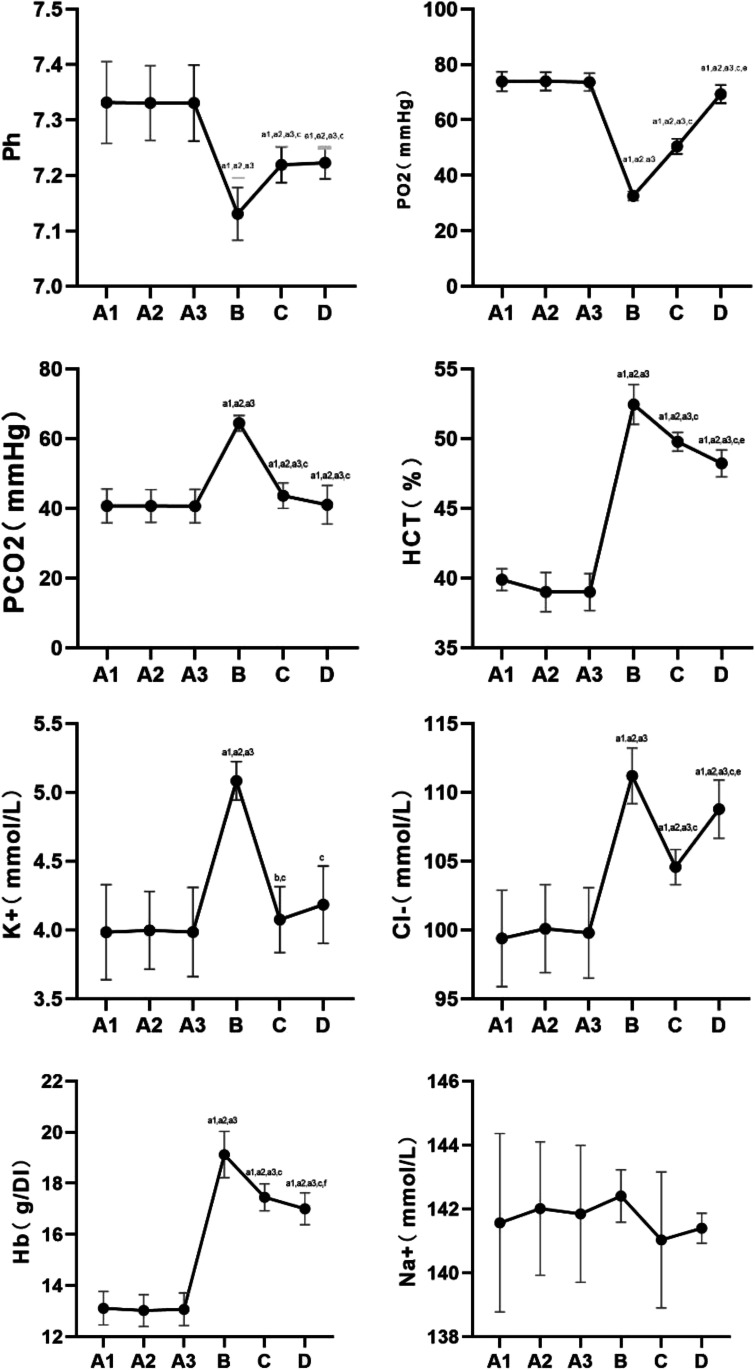
Analysis of rats’ arterial blood gas in each group. Note: compared with group A1, a1: *P* < 0.01, b1: *P* < 0.05; compared with group A2, a2: *P* < 0.01, b2: *P* < 0.05; compared with group A3, a3: *P* < 0.01, b3: *P* < 0.05; compared with group B, c: *P* < 0.01, d: *P* < 0.05; compared with group C, e: *P* < 0.01, f: *P* < 0.05

The results of blood gas analysis are shown in Fig. 2.

The results of blood gas analysis were not significantly different among groups A1, 2 and 3 (*P* > 0.05).

Compared with groups A1, 2 and 3, pH and PO^2^ levels were decreased in groups B, C and D, and the difference was statistically significant.

Compared with groups A1, 2 and 3, the levels of PCO^2^, HCT, K^+^, Cl^−^, and Hb in groups B, C and D were increased, and the difference was statistically significant. Na^+^ changes were not obvious in each group.

Compared with group B, pH and PO^2^ levels were increased in groups C and D, and the difference was statistically significant. Compared with group B, the levels of PCO^2^, HCT, K^+^, Cl^−^ and Hb in groups C and D were decreased, and the difference was statistically significant.

Compared with group C, the levels of PO^2^ and Cl^−^ were increased in group D, and the difference was statistically significant. Compared with group C, the levels of HCT and Hb in group D were significantly decreased.

Na^+^ changes were not obvious in each group.

### Serum Hormones

**Fig. 3:**
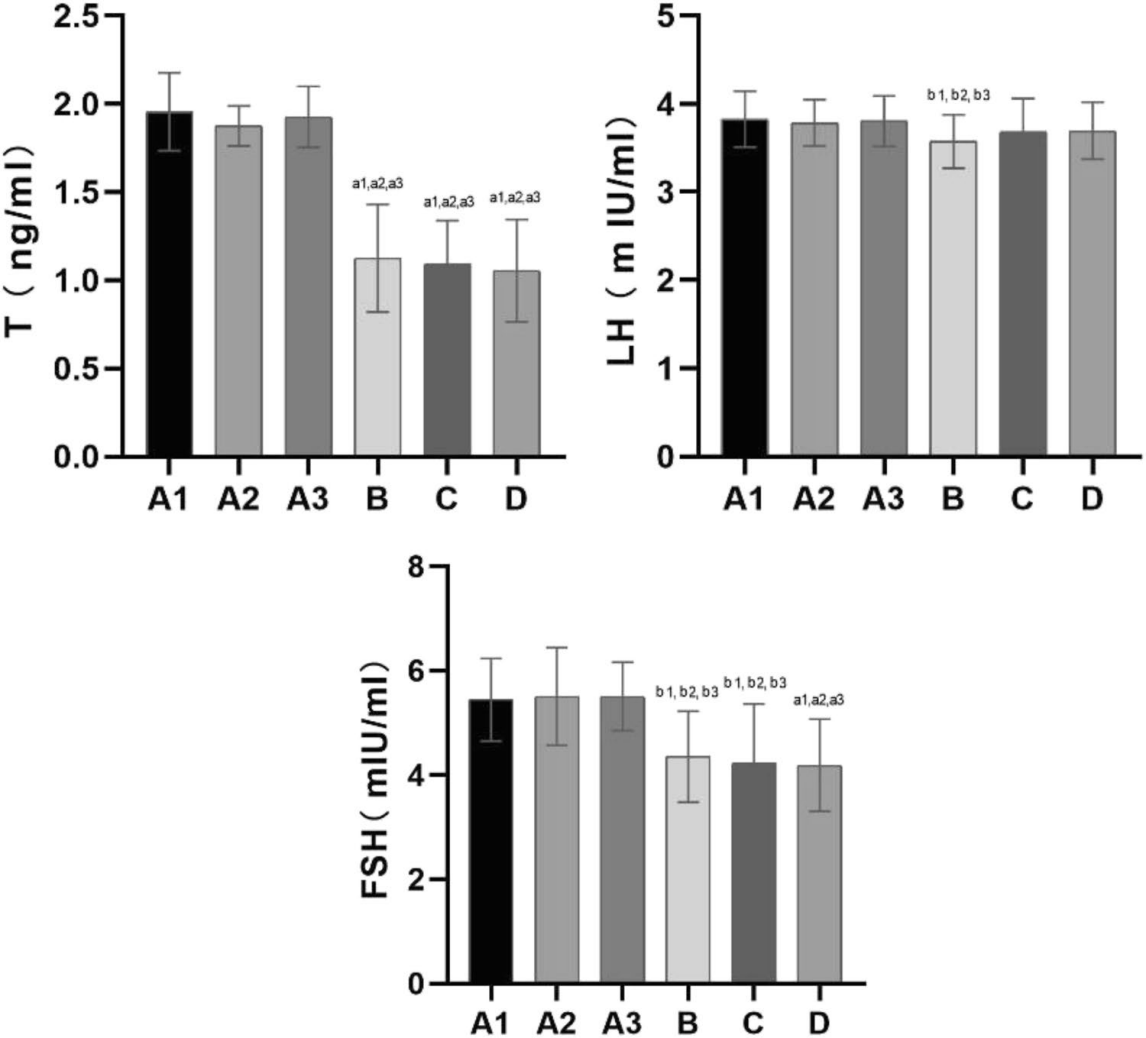
Serum levels of sex hormones such as T, LH and FSH in serum of rats in each group. Note: compared with group A1, a1: *P* < 0.01, b1: *P* < 0.05; compared with group A2, a2: *P* < 0.01, b2: *P* < 0.05; compared with group A3, a3: *P* < 0.01, b3: *P* < 0.05; compared with group B, c: *P* < 0.01, d: *P* < 0.05; compared with group C, e: *P* < 0.01, f: *P* < 0.05

The serum hormone results are shown in Fig. 3.

Serum hormone levels were not significantly different among groups A1, 2 and 3 (*P* > 0.05).

#### Testosterone (T)

Compared with groups A1, 2 and 3, the levels of T were decreased in groups B, C and D, and the difference was statistically significant (*P* < 0.01). The levels of T were not significantly different among groups B, C and D (*P* > 0.05).

#### Luteinizing Hormone (LH)

Compared with groups A1, 2 and 3, LH levels were decreased in group B, and the difference was statistically significant (*P* < 0.05). No differences were noted among groups B, C and D (*P* > 0.05).

#### Follicle-Stimulating Hormone (FSH)

Compared with groups A1, 2 and 3, FSH levels were decreased in groups B (*P* < 0.05), C (*P* < 0.05) and D (*P* < 0.01), and the difference was statistically significant. No significant differences were noted among groups B, C and D (*P* > 0.05).

### Testicular Tissue Morphology

**Fig. 4:**
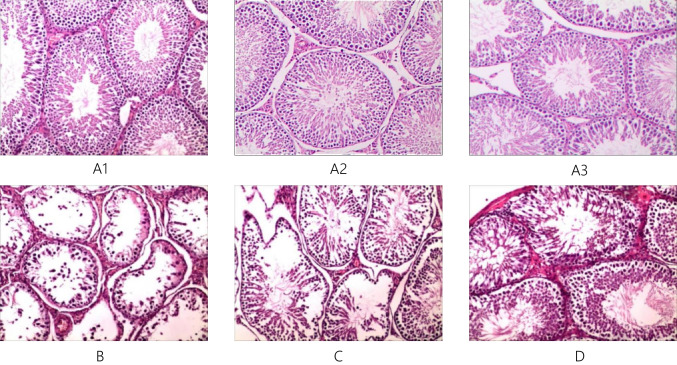
HE staining of testis tissue of each group of rats × 100. Note: group A1–3: A1, A2, A3; group B: B; group C: C; group D: D

The testicular tissue morphology of each group is shown in Fig. 4.

The testicular tissue morphological assessment results were not noticeably different among groups A1, 2 and 3.

Under a 100 × light microscope, we found that the basement membrane of seminiferous tubules in rat testis tissue was intact, exhibiting the whole process of spermatogenesis in groups A1, 2 and 3. Numerous sperm were present in the lumen, and no obvious signs of damage were noted. Approximately 5–7 layers of cells were arranged neatly, and regular cell polarity was noted in groups A1, 2 and 3. These results indicate that the cell structure and function were normal.

Compared with groups A1, 2 and 3, the basement membrane of the seminiferous tubules in the testis tissue of rats was atrophied, thinned, wrinkled and obviously stratified. In group B, vacuoles were clearly evident, and the number of sperm was very low. A few early spermatogenic cells were observed. The number of layers was reduced, and cell polarity disappeared. The cell arrangement was extremely disordered in group B, indicating that the cell structure and function were damaged.

Compared with groups A1, 2 and 3, the basement membrane of seminiferous tubules in the testis tissue of rats showed slight folds, atrophy and stratification. The number of sperm increased. Impaired spermatogenesis was not obvious, and mature spermatogenic cells were absent in groups C and D. In group C, 3–5 layers were noted, and cell polarity was visible. However, the pathological changes were more obvious than those in groups A1, 2 and 3. In groups C and D, 3–5 layers were observed, and cell polarity was visible. The cell arrangement was slightly disordered in groups C and D, but the pathological changes were more obvious than those in groups A1, 2 and 3.

Johnsen Score

The criteria of the Johnsen score are shown in Table 3.

**Table 3:** Johnsen score criteria

Score	Spermatogenesis level
10	The whole process of spermatogenesis is observed
9	Spermatogenesis was slightly impaired
8	Each seminiferous tubule has less than 5 sperm
7	Mature spermatogenic cells are absent. Most early spermatogenic cells can be observed
6	Early spermatogenic cells are reduced or missing and the spermatogenesis process is halted at the stage of spermatogenic cell formation
5	Many spermatocytes
4	Few spermatocytes and the spermatogenesis process are halted at the stage of primary spermatocytes
3	Only spermatogonia
2	No spermatogenic cells, only supporting cells
1	No vas deferens epithelial cells. Tubular sclerosis is noted

The morphological changes of seminiferous tubules, Sertoli cells and mesenchymal cells in the rat testis tissue were observed under a light microscope. The higher the scores, the better the spermatogenesis. The lower the scores, the worse the quality

**Fig. 5:**
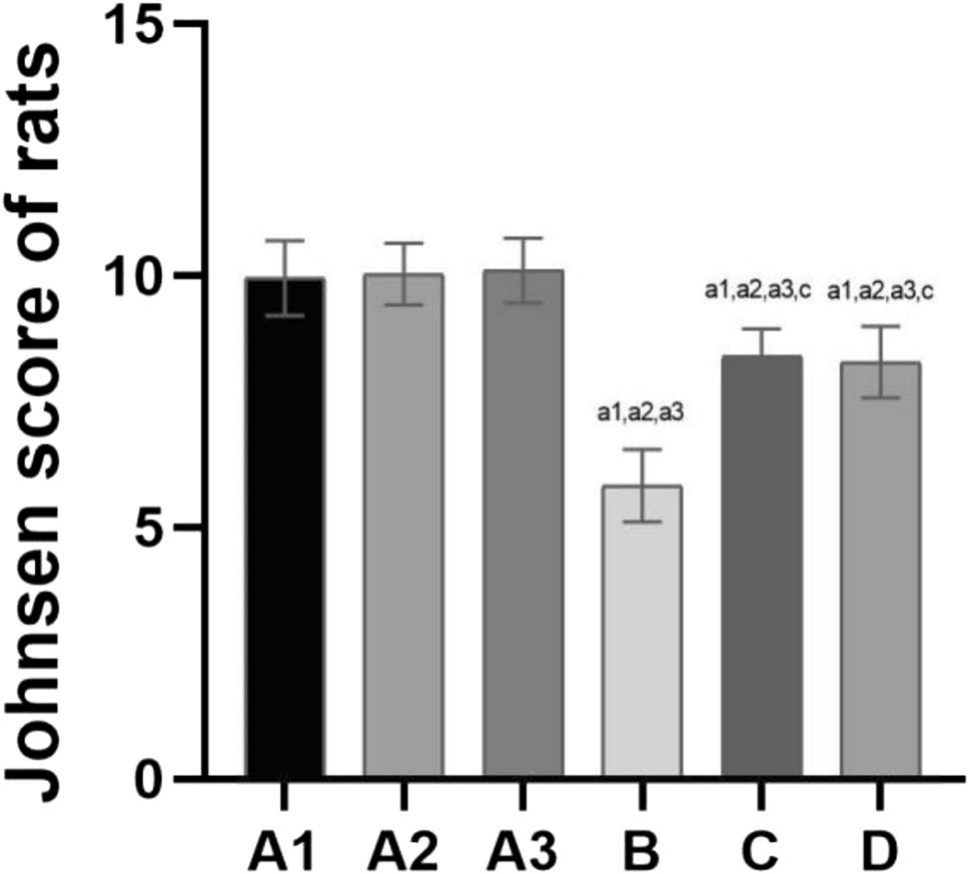
The results of Johnsen score of rats in each group. Note: compared with group A1, a1: *P* < 0.01, b1: *P* < 0.05; compared with group A2, a2: *P* < 0.01, b2: *P* < 0.05; compared with group A3, a3: *P* < 0.01, b3: *P* < 0.05; compared with group B, c: *P* < 0.01, d: *P* < 0.05; compared with group C, e: *P* < 0.01, f: *P* < 0.05

The results of the Johnsen score are shown in Fig. 5.

The Johnsen scores were not significantly different among groups A1, 2 and 3 (*P* > 0.05).

Compared with groups A1, 2 and 3, the Johnsen scores were decreased in groups B, C and D, and the difference was statistically significant (*P* < 0.01).

Compared with group B, the Johnsen scores were increased in groups C and D, and the difference was statistically significant (*P* < 0.01).

No difference was noted between groups C and D.

### Transmission Electron Microscopy (TEM) Results

**Fig. 6:**
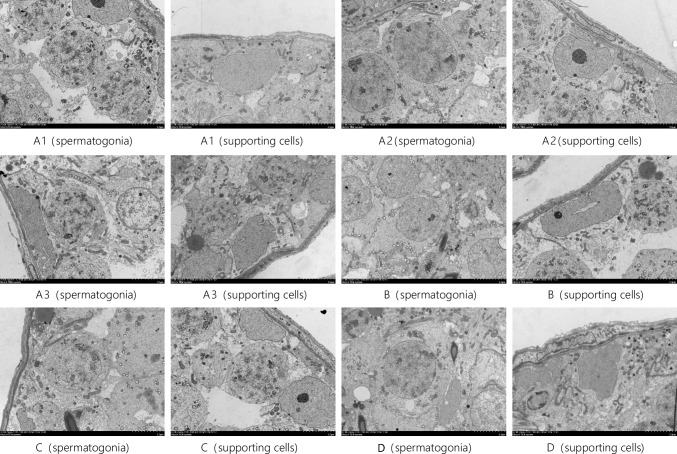
TEM observation results of spermatogonia and supporting cells in rat testis tissues of each group TEM × 5 μm. Note: group A: A1, A2, A3; group B: B; group C: C; group D: 2-D

Using TEM, we observed the ultrastructure of testis tissue in each group, including the spermatogonia and supporting cells. These results are presented in Fig. 6.

In groups A1, 2 and 3, normal supporting cells, mesenchymal cells, spermatogonia, primary spermatocytes and secondary spermatocytes were observed. The cell structure was clear. The basement membrane was intact, and a small number of lipid droplets were observed in the cells. Mitochondria were not swollen, and the endoplasmic reticulum of interstitial cells was not expanded.

In group B, a large number of vacuoles were present in the cytoplasm of supporting cells, where organelles exhibited obvious oedema and cavitation. The mitochondria were broken and swollen, and the lipids were abnormally increased. The nuclear membrane of interstitial cells was ruptured. Mitochondria in these cells were oedematous, and the endoplasmic reticulum was enlarged. The cytoplasm was locally absent and exhibited cavitation and heteronuclear staining. A small number of vacuoles were observed around the spermatogonia membrane, and the cell membrane was obviously damaged. The endoplasmic reticulum of the primary spermatocytes was slightly swollen. The cell membrane was not distinct, and nuclear heterochromatin increased. Large vacuoles formed around the secondary spermatocytes.

In group C, the Sertoli cells were slightly detached from the seminiferous epithelium, and the cell membrane and nuclear membrane structure remained clear. The mitochondria were slightly swollen and relatively normal. The mesenchymal endoplasmic reticulum was slightly expanded. The structure of spermatogonia in the nucleus and membrane was clearly observed.

In group D, the supporting cell structure was clear. In addition, a small number of vacuoles were observed in the cytoplasm, and the mitochondria were slightly swollen. The structure of interstitial cells, spermatogonia and primary spermatocytes was clearly noted. Specifically, basement membranes were intact, and mitochondria and endoplasmic reticulum were not swollen.

The spermatogonia and supporting cells (in groups B, C and D) exhibited different degrees of structural changes, and the most obvious changes were noted in group B. Both primary spermatocytes and secondary spermatocytes were structurally altered and destroyed in group B. Supporting cells were slightly detached from the seminiferous epithelium in group C. The structure of interstitial cells, spermatogonia and primary spermatocytes was clear in groups A1, 2 and 3. Numerous vacuoles were present in the cytoplasm of supporting cells in group B. The supporting cells were slightly detached from the seminiferous epithelium in group C. The structure and morphology of supporting cells in group D were similar to that noted in Group A.

### Rat Sperm Quality Test

**Fig. 7:**
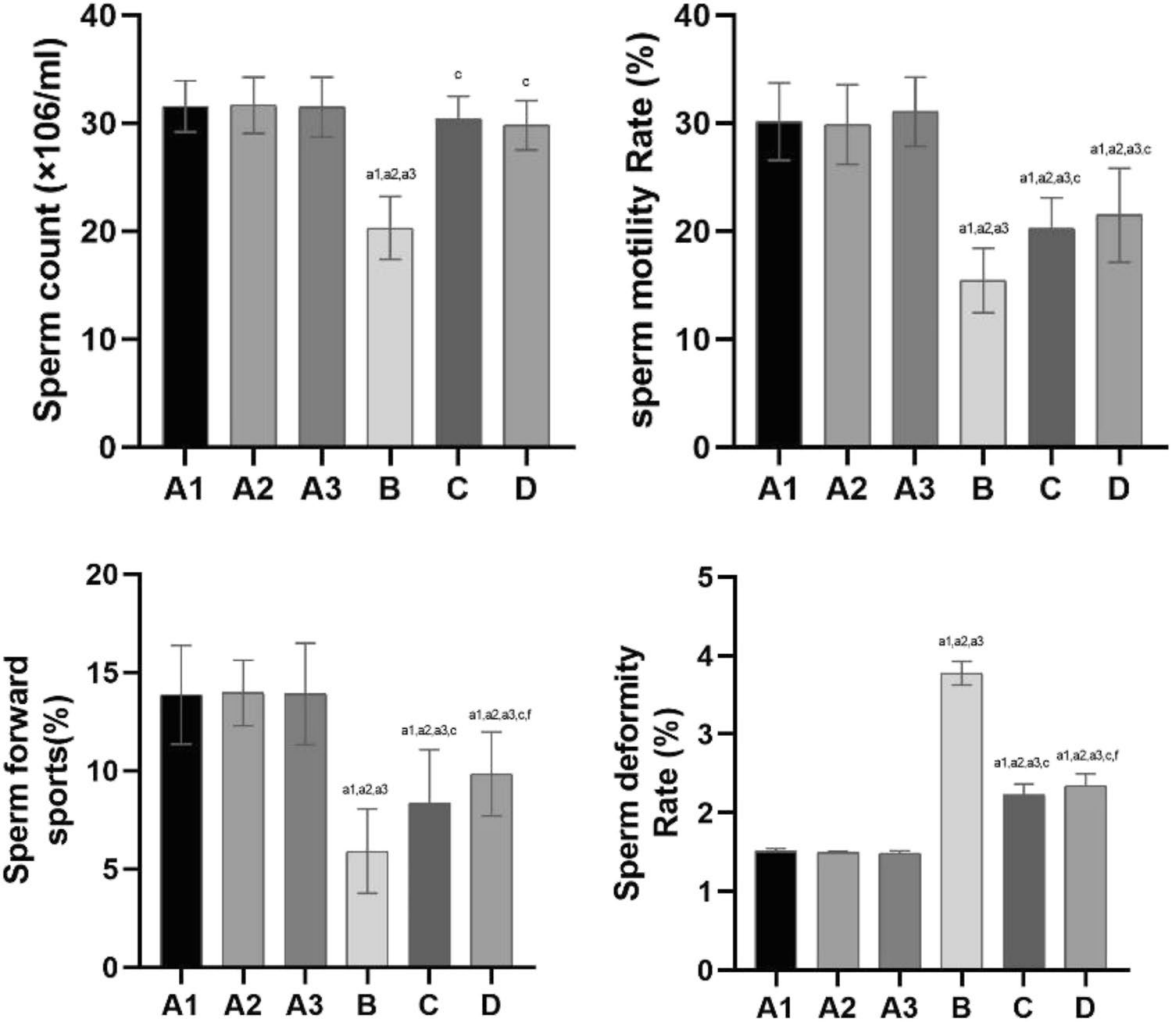
The results of rat sperm quality testing each group. Note: compared with group A1, a1: *P* < 0.01, b1: *P* < 0.05; compared with group A2, a2: *P* < 0.01, b2: *P* < 0.05; compared with group A3, a3: *P* < 0.01, b3: *P* < 0.05; compared with group B, c: *P* < 0.01, d: *P* < 0.05; compared with group C, e: *P* < 0.01, f: *P* < 0.05

The rat sperm quality test results are shown in Fig. 7.

The rat sperm quality test results showed no significant difference between groups A1, 2 and 3 (*P* > 0.05).

Compared with groups A1, 2 and 3, there was a significant decrease (*P* < 0.01) in the index of sperm motility or sperm forward movement and a significant increase (*P* < 0.01) in the index of sperm deformity in groups B, C and D.

Compared with every group, the index of sperm count was significantly decreased (*P* < 0.01) in group B.

Compared with group B, the index of sperm motility rate or sperm count was significantly increased (*P* < 0.01), and the index of sperm deformity was significantly reduced (*P* < 0.01) in groups C and D.

Compared with group B, the index of sperm forward movement was increased in groups C and D (*P* < 0.01). The index of sperm forward movement and sperm deformity differed (*P* < 0.05) between Group C and Group D.

### Oxidative Stress Damage Levels

**Fig. 8:**
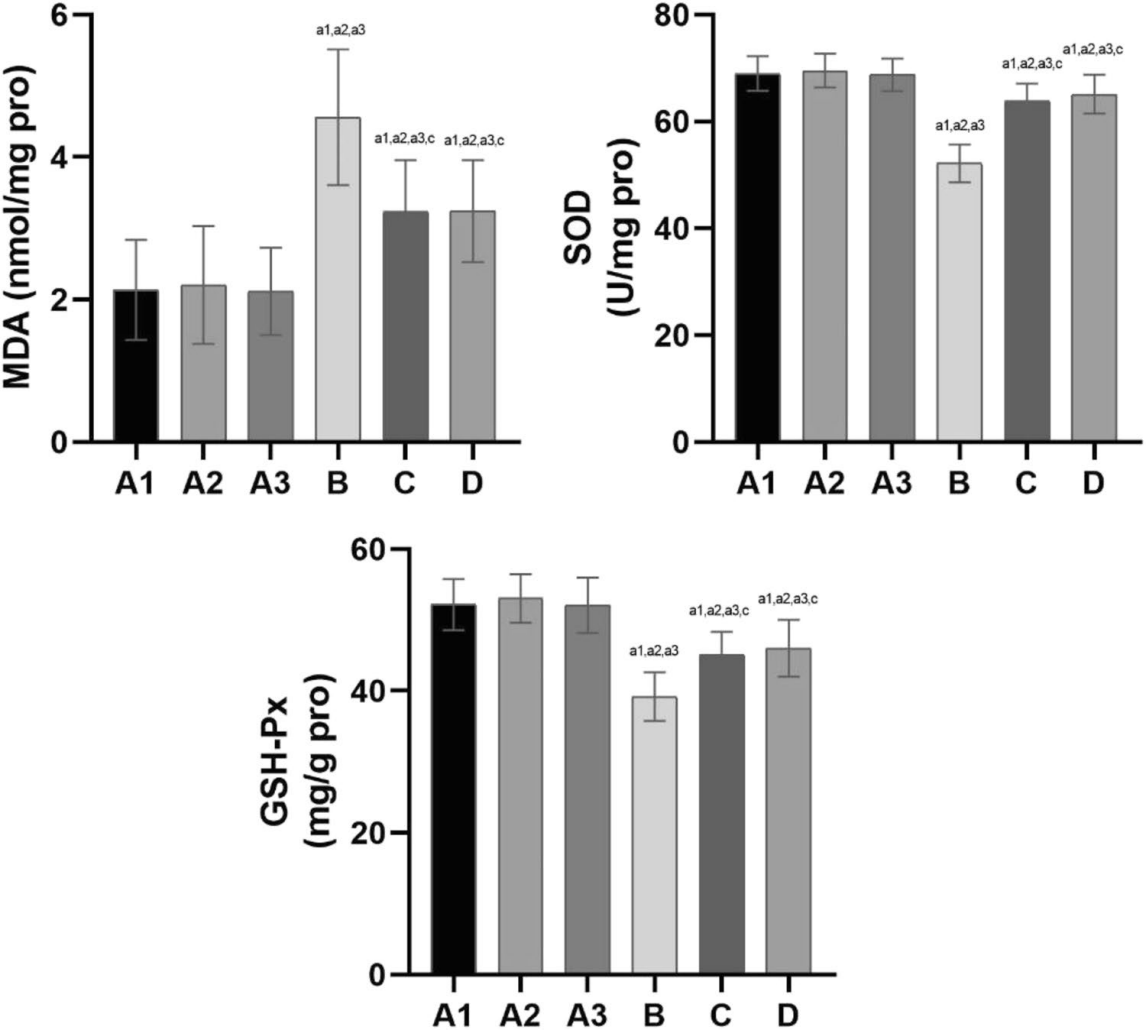
Effects of oxide and antioxidant activity in testicular tissue of rats in each group. Note: compared with group A1, a1: *P* < 0.01, b1: *P* < 0.05; compared with group A2, a2: *P* < 0.01, b2: *P* < 0.05; compared with group A3, a3: *P* < 0.01, b3: *P* < 0.05; compared with group B, c: *P* < 0.01, d: *P* < 0.05; compared with group C, e: *P* < 0.01, f: *P* < 0.05

Oxidative stress damage levels are shown in Fig. 8.

Oxidative stress damage levels were not significantly different among groups A1, 2 and 3 (*P* > 0.05).

Compared with groups A1, 2 and 3, the malondialdehyde (MDA) concentration was increased (*P* < 0.01) in groups B, C and D. In addition, superoxide dismutase (SOD) and glutathione peroxidase (GSH-Px) activity were decreased (*P* < 0.01).

Compared with group B, the MDA concentration was decreased (*P* < 0.01), and SOD and GSH-Px concentrations were increased (*P* < 0.01) in groups C and D. No significant differences in the MDA, SOD and GSH-Px concentrations were noted between groups C and D.

## Discussion

The term plateau refers to an area at least 3000 m above sea level, and its typical climatological characteristics are cold temperatures, low partial pressure of oxygen and high solar radiation. In recent years, numerous studies have shown that the high-altitude hypoxic environment causes nonnegligible damage to various organs and systems of the body. Low-oxygen exposure can trigger a series of complex chain reactions at the endocrine level, cell level and gene level. A large number of studies have shown that the high-altitude low-pressure and low-oxygen environments have a negative impact on the male reproductive system [4–6]. A high-altitude environment can reduce the level of sex hormones in males, damage testicular tissues and induce germ cell apoptosis [7, 8]. At present, the diagnosis of acute mountain sickness relies on the Lake Louise scoring standard or the domestic acute mountain sickness diagnostic standard [13, 14], which is mainly based on a series of symptoms that appear in a high-altitude hypoxic environment. Based on these standards, a quantitative score is used to diagnose acute mountain sickness.

### Basic Conditions of the Plateau Animal Model

Therefore, to perform research on high-altitude reproductive injury, the primary problem that needs to be solved is the generation of animal models of acute high-altitude reproductive system injury. However, once the animal model is established, it is necessary to select indicators to judge and evaluate whether the plateau animal model is successful given that it is impossible to quantify the symptoms of animals based on human standards. In addition, the choice of animal age is also very important because we need to select experimental animals that represent an age that is similar to human adults. The mature male reproductive system of rats is suitable for the study of reproductive system damage under simulated conditions of high altitude, low pressure and hypoxia. Therefore, 120 Wistar male rats housed in SPF conditions were selected for this study. These rats were 24 weeks of age (comparable to 18–20 years of age in humans), and their reproductive system is so well developed that they can complete autonomous mating [15, 16]. Some researchers [17] have found that the apoptosis rate of germ cells is the highest at 15 days, and the number of sperm in the epididymis is also the lowest at 15 days. These studies were designed to determine the effects on the rat’s reproductive system at an altitude of 5000 m at 5 days, 15 days and 30 days. Therefore, the following parameters were employed to generate the model: 6000 m and a 2 °C temperature difference between day and night to simulate a high-altitude environment (inside the cabinet) for 28 days. These conditions are advantageous to simulate a plateau environment based on high-altitude and low-oxygen exposure. This environment may reflect the damage of low-pressure and low-oxygen exposure to the male reproductive system. This model has been tested and exhibits good reproducibility.

### Research on the Influence of High Altitude with Low-Pressure and Low-Oxygen Conditions on Male Reproductive Function and Intervention Measures

Excessive production of active oxygen in the body, a decrease in the body’s antioxidant capacity or insufficient removal of active oxygen leads to an increase in ROS in the body and cause cell oxidative damage. The pathological process is called oxidative stress, which is also one of the main mechanisms of damage caused by high-altitude, low-pressure and low-oxygen environmental factors. Under normal physiological conditions, oxygen-free radicals in the organism are in a dynamic state of equilibrium: oxygen-free radicals are constantly produced and constantly eliminated. However, the influence of high-altitude factors may cause an increase in the body’s production of oxygen-free radicals or a decrease in the speed of scavenging oxygen-free radicals, which will cause oxidative stress [18–22]. Some studies have found that intermittent hypoxia leads to the following changes: a decrease in total antioxidant capacity, an increase in MDA serum levels, a decrease in the activity of antioxidants (SOD, GSH-Px and GSR) of the testis and epididymis and a significantly reduced number of epididymal sperm [23, 24].

At present, how to avoid or reduce the damage to sperm quality caused by a high-altitude environment has become an important topic worthy of in-depth study. Mitrović A. et al. [25] showed that hyperbaric oxygen therapy can significantly improve sperm motility in patients diagnosed with oligospermia. Some studies have shown that blueberry extract can exert antioxidative damage to reduce the damage that the hypoxic environment exerts on testicular tissue, but the quality of sperm has not been visually assessed [26]. The l-carnitine used in our experiments is a water-soluble antioxidant substance that has a good effect on the treatment of male varicocele, idiopathic oligoasthenospermia and reproductive inflammation. l-carnitine is very important to the male reproductive system. The highest concentration of free l-carnitine in males is noted in sperm, seminal plasma and epididymis, and l-carnitine is involved in sperm maturation, metabolism and flagella movement [11, 12, 27, 28]. l-carnitine can also regulate the metabolism of sugar, fat and protein in supporting cells and maintain the normal physiological functions of sperm by reducing the level of reactive oxygen species in sperm. Our findings have found that l-carnitine can reduce the damage to male sperm quality in a high-altitude environment but has no significant effect on serum sex hormone levels. l-Carnitine potentially improves sperm quality because it improves the ability of testicular tissues to cope with antioxidative damage and inhibits germ cell apoptosis. These mechanisms still need to be further studied. In our experiment, l-carnitine was adopted as a protective measure against adverse environmental factors of high-altitude hypotension and hypoxia. Various detection methods were used to evaluate the hypoxia status of animals in the plateau model before and after the administration of l-carnitine, including assessments of the organ index of testicular tissue, blood gas levels, serum hormone levels, testicular histopathology and sperm quality. Further information on our findings is presented below.

#### The Organ Index Refers to the Ratio of the Weight of Animal Organs to Body Weight

Any factor that affects the organ weight will cause this value to change. The value has a small fluctuation range under physiological conditions. However, under certain pathological conditions, excessive changes in organ weight due to organ congestion and oedema, hyperplasia and hypertrophy, atrophy or degeneration will occur. The organ index is used as a reference index to reflect organ damage [29, 30]. Some experimental results show that the organ index of the testicular tissue is lower in rats in a simulated plateau environment with low-pressure and low-oxygen environment compared with the control group, indicating that the low pressure and high altitude may cause damage to the rat’s testicular tissue and result in atrophy or degeneration or a decreased organ index [4, 31–35]. In our experiment, the organ indices of groups B, C and D all decreased (*P* < 0.01, the difference was statistically significant), whereas no effect was noted in group A. This result once again verified the impact of high-altitude environmental factors on testicular tissue damage. After the administration of l-carnitine, the organ indices of group C and group D were increased (*P* < 0.01, the difference was statistically significant) compared with group B. This finding demonstrates that l-carnitine may have a positive effect on rat testicular tissue under a simulated high-altitude environment and indirectly indicates that l-carnitine may effectively eliminate ROS, participate in cell energy metabolism, inhibit apoptosis and protect sperm DNA integrity [36]. Compared with group C, the organ index of group D was increased (*P* < 0.05, the difference was statistically significant), indicating that high-dose l-carnitine may have a greater positive effect on rats in a simulated high-altitude environment.

#### Blood Gas Analysis is One of the Main Methods Used to Evaluate the Body’s Hypoxic State and Acid–Base Imbalance

Blood gas analysis is often used in medicine to judge whether the body has an acid–base imbalance and determine the degree of hypoxia [37]. In our experiment, some indicators in the blood gas analysis of the plateau animal model changed when the simulated altitude was 6000 m. Specifically, pH and PO^2^ values decreased, whereas PCO^2^, HCT, K^+^, Cl^−^, and Hb values increased. The analysis data showed that the higher the simulated altitude, the lower the partial pressure of oxygen and the more obvious the increase in carbon dioxide. Therefore, blood gas analysis can be used to evaluate hypoxia in the plateau animal model in the early stage of hypoxia.

In this experiment, arterial blood gas results indicated different degrees of hypoxia in groups B, C and D animals housed in a hypobaric hypoxic animal chamber: decreased PO^2^, increased PCO^2^, increased Hb or HCT, and elevated K^+^ and Cl^−^ levels. After supplementation with l-carnitine, especially in group D (high-dose), the hypoxic condition was improved to some extent. Increased PO^2^ and decreased PCO^2^ were noted in groups B, C and D compared to Group A, suggesting that the hypoxic condition was improved after the administration of l-carnitine.

The following potential mechanism is proposed: l-carnitine prolongs the ability of endothelial cells to regulate blood flow during ischaemia, and an increase in serum carnitine concentration enhances capillary endothelial function [38]. The role of early l-carnitine supplementation has been examined in substrate metabolism and in acute exercise performance in prior research. The effects of l-carnitine on endothelial function and nitric oxide release have been demonstrated in animal studies and human clinical trials [39]. In these studies, supplementation with l-carnitine reduced structural and biochemical muscle damage and facilitated tissue repair by protecting against carnitine deficiency in endothelial cells, thereby ameliorating blood flow and oxygen supply [40]. Some studies have shown that ion channels (such as ATP-dependent K^+^ channels and Ca^2+^ -activated K^+^ channels) play a role in adapting to changes in the high-altitude hypoxic environment by altering serum ion levels. According to the literature, l-carnitine may also be involved in the regulation of some ion channels [41–43]. However, the related mechanism of blood oxygen content and serum ions in a hypoxic environment must be studied further.

#### Serum Sex Hormone Levels Combined with Sperm Quality Are Important Indicators to Assess Male Reproductive Ability

Recently, controversial research results regarding the influence of a plateau environment on serum sex hormone levels have been reported. Numerous serum sex hormones can be assessed. In our experiment, only three indicators, namely, serum LH, FSH and T levels, were selected given that these hormones are closely related to male reproductive ability. Studies have demonstrated that LH levels increase significantly during the initial stage of entering the plateau and then reach the standard normal level. However, living on the plateau for a long time does not have an impact on LH and FSH levels [44, 45]. Tafuri A et al. [46] observed the serum sex hormones of people who moved to the plateau and then returned to the plains, and the results showed that exposure to high altitude affected the hormonal axes. Their studies have also shown that the effect seemed notably pronounced for the hypothalamus-pituitary gonadal axis, which was suppressed after high-altitude exposure. He J et al. demonstrated that hypoxia at high altitude causes adverse effects on sperm quality and reproductive hormones, and these effects are reversible [47]. In our experiment, rats were reared in the hypobaric oxygen chamber for 28 days to simulate a high-altitude environment. Serum T levels were reduced, but the reduction in serum FSH levels observed in our study differed from the results of previous studies. The administration of l-carnitine in our experiment had no significant effect on serum FSH, LH and T levels in the plateau animal model. The reproductive endocrine system is complex, and various factors can be affected. The study of the influence of a high-altitude environment on serum sex hormones may require in-depth research on the molecular mechanism.

#### The Effect of High-Altitude Hypoxic Environmental Factors on Sperm Quality and the Positive Effect of l-Carnitine

Hypoxic conditions will cause blood to redistribute to important organs, such as the brain and heart, resulting in reduced blood flow in the testicles. In addition, sperm production consumes a large amount of oxygen. However, the high-altitude hypoxic environment cannot meet the oxygen demands of spermatogenesis. These low-pressure and low-oxygen conditions will damage sperm quality. The hypoxia pathway is also highly related to the pathological mechanism of male infertility caused by varicocele. The excess production of ROS is one of the main molecular causes that contributes to varicocele-related male infertility, in which MIR210HG and MLLT4-AS1 (hypoxia-related lncRNAs) also showed significant positive correlations with ROS and negative correlations with sperm count and motility [48]. Dan Zeng et al. [49] also demonstrated that the average sperm concentration of male residents of Lasa at reproductive age was lower than those in the plains. Some scholars also believe that the plateau environment negatively affects the quality of male sperm, and these effects increase as the altitude increases [50]. Studies by Lan Tian, Lin Kai and others [51–53] show that the plateau environment can reduce the concentration of male sperm, weaken vitality and increase the deformity rate. Based on the above research and analysis, it has been found that the high-altitude, low-pressure and low-oxygen environment may have a significant impact on the health of the male reproductive system, resulting in a decrease in the number of sperm, reductions in sperm motility and viability, an increase in the rate of sperm abnormalities, an increase in germ cell apoptosis and increased disorders of sex hormone secretion [54, 55]. The results of these studies are consistent with the results of our experimental study. In our study, sperm motility, deformity rates and the sperm counts of rats from the high-altitude model were assessed, and a preliminary analysis of sperm morphology was performed. The results demonstrate that sperm viability and sperm count decrease and the sperm deformity rate increases as the altitude increases, and the effects are most obvious at an altitude of 6000 m. The higher the altitude is, the more obvious the damage. After the administration of l-carnitine, we found that the high- and low-dose l-carnitine groups also exhibited significantly improved sperm motility rates, sperm counts and sperm abnormality rates in a hypoxic environment, and the high-dose group also improved the percentage of forward movement in sperm.

#### Histopathological Examination of the Testis to Directly Assess the Seminiferous Tubules of the Testis as the Gold Standard for Assessment of Spermatogenic Function of the Testis

Compared with group A, the basement membrane of the seminiferous tubules of the testis tissue in group B was atrophied, thinned, wrinkled and stratified based on HE staining. Obvious vacuoles, very small amounts of sperm, a small number of early spermatogenic cells, extremely disordered cell arrangement, reduced cell layer number, the disappearance of cell polarity and a reduced Johnsen score were observed. The following phenomena were observed based on TEM analysis in group B: a large number of vacuoles in the cytoplasm, obvious oedema and cavitation of organelles, increased levels of abnormal lipids, rupture of the nuclear membrane of interstitial cells, mitochondrial oedema, partial absence of cytoplasm, increased nuclear heterochromatin, expansion of endoplasmic reticulum, obvious damage to the spermatogonia membrane, a few vacuoles near the membrane, endoplasm of primary spermatocytes, insignificant swelling of the endoplasmic reticulum, the lack of a defined cell membrane, increased nuclear heterochromatin and the formation of large vacuoles next to secondary spermatocytes. These changes in the ultrastructure of rat testicular tissues indicated that the simulated high-altitude, low-pressure and hypoxic environment affects the tight junctions between rat Sertoli cells, mesenchymal cells and spermatogonia. These effects lead to obvious cell damage, further affecting the spermatogenic function of the rat testis.

However, HE staining showed slight folding, shrinking and delamination of the basement membrane of seminiferous tubules in the rat testicular tissue, an increasing amount of sperm, insignificant damage to spermatogenesis, a lack of mature spermatogenic cells, a slightly disordered and sparse cell arrangement with 3–5 layers and visible cell polarity in groups C and D. The Johnsen scores of groups C and D are higher than that of group B but still lower than that of Group A.

TEM revealed that Sertoli cells were slightly detached from the seminiferous epithelium, where the cell membrane and nuclear membrane structure were still clear. In addition, mitochondria were slightly swollen, and the mesenchymal endoplasmic reticulum was slightly expanded. The spermatogonia nuclear membrane structure was clear, and mitochondria were relatively normal in groups C and D. There were no significant differences that were noted between groups C and D, indicating that l-carnitine has a good protective effect on male reproductive damage caused by low-pressure and low-oxygen environments.

The protective effect of l-carnitine against the damage to the male reproductive system caused by the simulated plateau environment may be related to its antioxidant effect. Some studies have shown that mitochondrial oxidative phosphorylation is an important system involved in energy production in various cells to maintain cell energy and integrity. In this system, l-carnitine is essential for the uptake of fatty acids into mitochondria. Fatty acid oxidation needs to pass through the mitochondrial membrane to enter the mitochondria and uses l-carnitine to transport fatty acids into the mitochondria for the subsequent step of β-oxidation [36], thereby increasing energy supply and maintaining cell integrity. l-carnitine also binds to acyl residues derived from the intermediate metabolism of amino acids and facilitates their function as scavengers. As an antioxidant, the concentration of l-carnitine in seminal plasma positively correlates with semen quality. l-Carnitine can prevent oxidative stress by regulating cellular respiration, nitric oxide levels and the activity of enzymes involved in antioxidative damage [56]. Higher concentrations of l-carnitine have a progressive inhibitory effect on β-oxidation, which is specific to l-carnitine [57]. However, given that the calcium chelator activity of l-carnitine may induce cell damage and decrease serum calcium levels, the clinical benefits or improvements in in vitro fertility will not be achieved by the overuse of antioxidants at very high doses. Researchers [58, 59] have also confirmed that l-carnitine can regulate the metabolism of cellular fat, sugar and protein, reduce excessive ROS generation, remove excess ROS and maintain the normal physiological function of sperm. Chavoshi Nezhad N et al. [58] showed that the use of antioxidants (l-carnitine and coenzyme Q10) in vitro could improve sperm motility and reduce the percentage of sperm DNA fragmentation in the clinical laboratory setting during ART procedures. Yang K et al. [59] discovered that an extender containing l-carnitine could improve sperm quality and increase the number of sperm bound to the zona pellucida. The use of exogenous antioxidants (l-carnitine) is a key strategy to alleviate time-dependent structural and biochemical damage to sperm caused by the inappropriate formation of ROS in liquid-preserved boar semen. Antioxidants may act as suitable sperm preservation agents given their beneficial effect on preserving sperm quality [60].

#### l-carnitine Administration Significantly Reduces the Oxidative Stress Response and Enhances Antioxidant System Activity in a Simulated Hypobaric Hypoxic Environment

Under normal conditions, active antioxidant enzymes can effectively eliminate ROS, maintain the balance of the oxidation-antioxidant system and promote the development and differentiation of germ cells. However, abnormalities may cause spermatogenesis disorders [61]. SOD and GSH-Px are antioxidant enzymes, and MDA is an indicator of peroxidation. In our experiment, MDA levels increased and levels of the antioxidant SOD decreased in the testis tissue in group B, indicating that the production and removal of oxygen-free radicals in the testis tissue of rats in a simulated high-altitude environment were unbalanced. To assess the protective mechanism and effect of l-carnitine on damage to the male reproductive system in a simulated high-altitude hypobaric hypoxic environment, we measured SOD, GSH-Px and MDA levels in the testis tissue of each group of rats. The results showed that MDA levels in group B were the highest compared with the other groups, indicating that ROS generated oxidative stress and damage. Levels of the antioxidants SOD and GSH-Px were decreased in group B, suggesting that antioxidant activity was increased and antioxidant enzyme consumption was increased. These results indirectly indicate that the oxidative damage in rats increased. Based on a statistical analysis of the data in groups C and D, MDA levels were significantly lower than those in group B, indicating reduced ROS generation. However, the levels of SOD and GSH-Px (members of the enzymatic antioxidant system) were increased in groups C and D compared with group B. In rats exposed to a simulated plateau environment with low pressure and a low oxygen, l-carnitine significantly reduces the oxidative stress response and enhances antioxidant system activity. These findings also demonstrate that the antioxidant effects of l-carnitine are reliable.

In summary, based on the successful construction of the plateau model of rat reproductive system injury, we conducted an exploratory study on the protective effects of l-carnitine on rat reproductive system damage in a simulated high-altitude environment. Under the simulated plateau altitude of 6000 m, partial pressure of oxygen values, sex hormone levels, sperm motility rates, sperm deformity rates and histopathological and morphological features of testicular tissues are obviously altered, suggesting that the environment at an altitude of 6000 m may have a significant impact on the function of the rat's reproductive system.

The administration of l-carnitine reduces the damaging effects of in a high-altitude environment on sperm quality in male rats. However, l-carnitine does not have a significant effect on serum sex hormone levels. Improved sperm quality may improve the ability of testicular tissues to resist oxidative damage and inhibit germ cell apoptosis, but these mechanisms still require further in-depth study.
